# The molecular pathology of p53 in primitive neuroectodermal tumours of the central nervous system

**DOI:** 10.1038/sj.bjc.6600151

**Published:** 2002-04-08

**Authors:** A S Y W Burns, E Jaros, M Cole, R Perry, A J Pearson, J Lunec

**Affiliations:** Cancer Research Unit, Medical School University of Newcastle, Newcastle Upon Tyne, UK; Neuropathology, Newcastle General Hospital, Newcastle Upon Tyne, UK; Department of Statistics, University of Newcastle, Newcastle Upon Tyne, UK; Department of Child Health, Royal Victoria Infirmary, Newcastle Upon Tyne, UK

**Keywords:** central primitive neuroectodermal tumours, p53 protein, DNA sequencing, Waf-1, Bax, Bcl-2

## Abstract

One hundred and one pre-treatment primary central primitive neuroectodermal tumours were analysed for the expression of p53 protein by immunohistochemistry using the monoclonal antibody DO-7. The staining intensity was classified into four groups: strong, medium, weak and negative and strong staining intensity was associated with the poorest survival. DNA sequencing of the p53 gene was performed in 28 cases representing all four staining groups. Mutations were found in only three of the strong staining tumours suggesting that DNA mutations were not common events and that in the majority of the tumours with over-expressed p53, the protein was likely to be wild-type. Results of immunohistochemistry showed a significantly positive relationship between the expression of p53 and Bax and Bcl-2 proteins, but not Waf-1. Multivariate analyses supported the prognostic value of p53 immunostaining in central primitive neuroectodermal tumours and also of age and gender of patients.

*British Journal of Cancer* (2002) **86**, 1117–1123. DOI: 10.1038/sj/bjc/6600151 www.bjcancer.com

© 2002 Cancer Research UK

## 

Primitive neuroectodermal tumours of the central nervous system (cPNETs) account for 6–8% of all brain tumours and for 12–25% of paediatric brain tumours (Roberts *et al*, 1991). These malignant tumours frequently occur between the ages of 3 and 12 and between 50–70 (de Vita *et al*, 1982) and are sensitive to radiotherapy (Chang *et al*, 1969). With advances in radiotherapy, neurosurgery and better supportive care, overall survival rates have been greatly improved in the last two decades from 24 to 82%. Specifically, 70% of patients survive for up to 5 years (Cervoni and Cantore, 1995). However, cranio-spinal radiotherapy causes undesirable long-term sequelae in the young age group, including stunted growth and loss of intellectual function. The use of adjuvant chemotherapy in permitting the reduction of radiotherapy dose is currently being investigated.

A wide range of clinical and biological factors have been studied for their effects on patient survival in the hope of identifying high risk and low risk groups in order to tailor aggressive treatment regimens according to patient prognosis. Among clinical factors studied are patients' age and gender extent of tumour resection and presence of metastasis at diagnosis (Tomita and McLone, 1986; Cervoni and Cantore, 1995; Sure *et al*, 1995; Weil *et al*, 1998). The biological disease features studied include expression of p53, loss of heterozygosity (LOH) of chromosome 17, differentiation markers and DNA ploidy (Yasue *et al*, 1989; Saylors *et al*, 1991; Badiali *et al*, 1993; Wang *et al*, 1998). Thus far, a reliable marker of disease outcome remains to be identified, although it has been noted that the most frequently reported feature in cPNETs is LOH of chromosome 17p, in particular the region containing the p53 gene locus and further telomeric to it (Thomas and Raffel, 1991; Albrecht, 1994). The p53 gene has been reported to be mutated in most cancers (Levine, 1997), and mutant p53 proteins often accumulate in the nucleus because their altered conformation endows them with a property to escape normal degradation.

Our group has previously reported the prognostic value of the immunohistochemical staining intensity of p53 protein in a series of 88 cPNETs (Jaros *et al*, 1993). In the previous study, immunohistochemistry for p53 was performed without any form of antigen retrieval and high intensity of immunohistochemical staining was significantly associated with poor patient survival. In the current study, immunohistochemistry was performed with microwaving for 85 cases of the original series of cPNETs and 16 new cases. The relationship between the intensity of p53 staining and patient survival was also investigated. As the accumulation of p53 protein in cells could be the result of normal reaction to genotoxic stress by wild-type p53 or of mutations in the p53 gene (Hainaut, 1995) not distinguishable by DO-7 immuno-staining, the coding regions of the p53 gene were sequenced in selective samples to establish the p53 gene status. The expression of other proteins involved in the p53 pathway as downstream mediators of p53 function, i.e. Waf-1, Bax and Bcl-2 (El-Deiry *et al*, 1993; Miyashita *et al*, 1994a,b; Miyashita and Reed, 1995), was also studied by immunohistochemistry, and their prognostic value was investigated by survival analyses. Finally, the expression of the above proteins, patients' age, sex and year of diagnosis were entered into a multivariate analysis.

## MATERIALS AND METHODS

### Patient samples

One hundred and one patients presenting with cPNETs (including 80 medulloblastomas) in the Northern region between 1963 and 1997 were studied, including 85 from the previous study mentioned above (Jaros *et al*, 1993) and 16 new cases. Patient samples were obtained from surgery, formalin-fixed and embedded in paraffin blocks. All were pre-treatment tissues, i.e. none of the patients had pre-operative radiotherapy or chemotherapy. All patients had attempted surgical removal of the primary tumour which resulted in either partial or total resection. The majority had craniospinal radiotherapy with a boost to the centre of the primary tumour, and some of those treated in the 1990s also received chemotherapy. Diagnosis of the tumours was made by the consultant neuropathologists in post, and the majority reviewed by two neuropathologists. For the purpose of the study, all pathology was reviewed by Professor Perry. Patient information was extracted from a combination of sources: patients' clinical notes obtained from the Medical Records, Newcastle General Hospital, and Middlesbrough General Hospital, and the ‘Children and Young Persons' Malignant Disease Registry’ at the Sir James Spence Institute of Child Health, Royal Victoria Infirmary (RVI), Newcastle Upon Tyne.

### Immunohistochemistry: antibodies and controls

A standard immunohistochemical staining method, described previously (Jaros *et al*, 1993), was performed in conjunction with an antigen retrieval technique. Prior to incubation with the primary antibody, sections were placed in citrate buffer and microwaved at high power for 10 min. Positive and negative controls were set up with every experiment. The same procedures were used for samples and the positive and negative controls, except that the primary antibody was omitted from the negative control. The antibodies used for p53, Waf-1, Bax and Bcl-2 were DO-7 (Novocastra) AB-1 (Oncogene), 13666E (Pharmingen) and 124 (Dako) and diluted at 1 : 50, 1 : 20, 1 : 1200 and 1 : 100, respectively. The control tissue used for p53 was obtained from a glioblastoma multiforme (GBM) paraffin-embedded tumour block. Sections from this block had previously been found to stain positive for DO-7 (Jaros *et al*, 1993). The control tissue used for Waf-1 staining was breast tumour tissue, and that for both Bax and Bcl-2 was tonsil tissue. The labelling index (LI) for each antibody was defined as the percentage of the number of positive nuclei (p53 and Waf-1), cytoplasm (Bcl-2) or both (Bax) in 1000 tumour cells. The staining intensity (SI) was defined with reference to the positive control (strong) and could be categorised into four groups: strong (A), medium (B), weak (C) and negative (D). Both LI and SI were assessed by two observers using a light microscope, with 95% consensus.

### DNA extraction from paraffin-embedded samples, PCR amplification and direct sequencing of exons 2–11 of the p53 gene

Genomic DNA was extracted from eight cases from p53 group A, 11 from groups B and C, and nine from group D for DNA sequencing. The procedures for extracting DNA from paraffin-embedded tissues, PCR amplification and direct sequencing of exons 4–9 of the p53 gene from genomic DNA, using the biotin method for purification of a single-stranded template, and the sequences of the primer pairs, have previously been described in detail (Challen *et al*, 1992). The sequences for primers corresponding to exons 2, 3, 10 and 11, and a new pair for exon 4, are described in Table 1

**Table 1:**
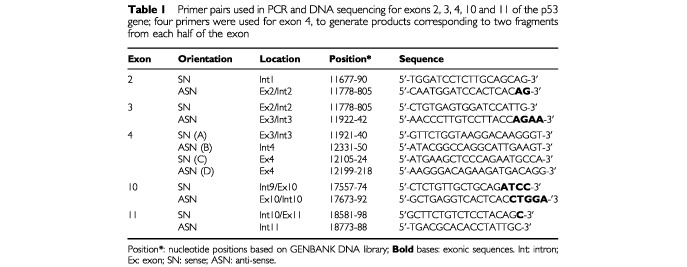
Primer pairs used in PCR and DNA sequencing for exons 2, 3, 4, 10 and 11 of the p53 gene; four primers were used for exon 4, to generate products corresponding to two fragments from each half of the exon

.

### Labelling of PCR product for exon 5 of the p53 gene

This method was applied to all samples to detect the presence of an 18 bp deletion in exon 5 which was identified in one sample during sequencing. A radiolabelled nucleotide α-^32^P-dATP was incorporated into the PCR products to enable visualisation by acrylamide gel electrophoresis and subsequent phosphorimaging or autoradiography. Slight adjustment was made to the dNTP (deoxynucleotide triphosphate) contents in the PCR mixture, to maintain the dATP concentration within adequate limits for reaction efficiency and fidelity during PCR.

### Survival analyses

The Kaplan–Meier method (Graphpad Prism) was used to estimate survival probability as a function of time, and the log-rank test to examine differences in survival between subgroups. Multivariate analysis for p53 SI and LI, age, sex and year of diagnosis (YOD), was performed by Cox's regression using the forward stepwise selection method (SPSS statistical software package).

## RESULTS

### Immunohistochemical analysis

#### Detection of p53, Waf-1, Bax and Bcl-2 proteins in normal cerebellar tissue

**Figure 1:**
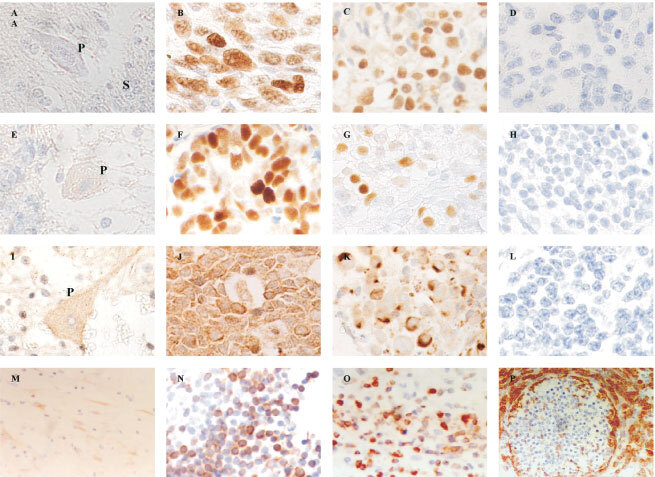
(**A**) Negative p53 staining in a Purkinje cell (P), and in the synaptic areas (S) in the space surrounding the granule cells. (**B**) Positive p53 staining in glioblastoma multiforme used as positive control. (**C**) Positive p53 immunostaining in cPNET. (**D**) Negative staining for p53 in cPNET. (**E**) Weak Waf-1 staining was observed in Purkinje cell (P) while adjacent granule cells were negative. (**F**) Strong nuclear staining of Waf-1 in breast carcinoma cells used as positive control. (**G**) Strong nuclear staining of Waf-1 in cPNET. (**H**) cPNET negative for Waf-1. (**I**) Moderate staining of Bax in the cytoplasm and dendrites of Purkinje cell. (**J**) Strong cytoplasmic staining of Bax in tonsil tissue (positive control). (**K**) Positive cytoplasmic staining of Bax by 13666E in cPNETs. (**L**) Negative staining of Bax in cPNET. (**M**) Postive Bcl-2 staining in the molecular layer of normal cerebellum. (**N**) Positive cytoplasmic staining of Bcl-2 in tonsil (control tissue). (**O**) High LI of Bcl-2 staining in cPNET. (**P**) Bcl-2 staining mainly outside pale islands in desmoplastic medulloblastoma.

p53 and Waf-1 were not detectable in normal cerebellar tissues present in 10 tumour sections (Figure 1A

, E). Bax was detected in normal cerebellar tissues, mostly in the cytoplasm (Figure 1I). Normal astrocytes were negative whereas reactive astrocytes were moderately stained. The staining of Bcl-2 in normal cerebellar tissue was generally weak, with Purkinje cells and other neurons being weakly or not stained at all (Figure 1M).

#### Detection of p53, Waf-1, Bax and Bcl-2 proteins in control tissue

Detection of p53, Waf-1, Bax and Bcl-2 in the corresponding control tissue was positive and strong, as expected (Figure 1B, F, J, N).

#### Detection of p53, Waf-1, Bax and Bcl-2 proteins in cPNETs

In cPNETs both positive and negative staining were observed for all four proteins (Figure 1C, G, K, O). A range of SI and LI was observed, but the majority of tumours showed weak to moderate staining. Both p53 and Waf-1 staining was localised to the nucleus, while Bax staining was observed in nucleus and cytoplasm. The staining of Bcl-2 was localised to the cytoplasm. Examples of negative staining for p53, Waf-1, Bax and Bcl-2 are shown in Figures 1(D, H, L, P) respectively. The distribution of the number of cPNETs in each LI and SI category for each protein is shown in Table 2

**Table 2:**
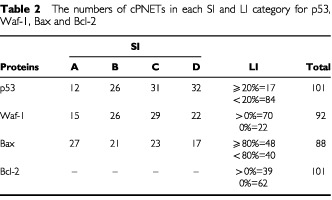
The numbers of cPNETs in each SI and LI category for p53, Waf-1, Bax and Bcl-2

and Figure 2

**Figure 2:**
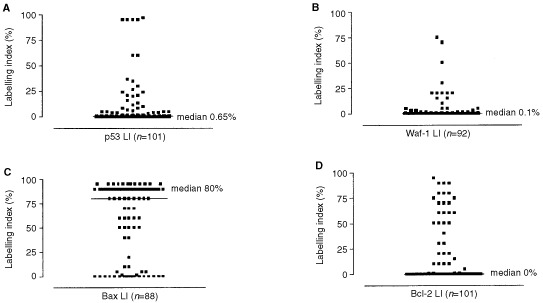
Distribution for LI for p53 (**A**), Waf-1 (**B**), Bax (**C**) and Bcl-2 (**D**) proteins in cPNETs.

(A–D).

### DNA sequencing of the p53 gene and PCR/direct labelling

**Figure 3:**
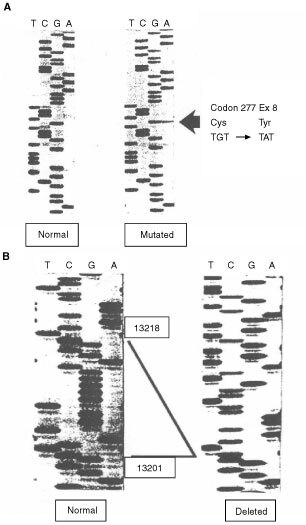
Mutations found in the p53 gene in two cPNETs by DNA sequencing. (**A**) Missense mutation at codon 277 in exon 8 in one cPNET. The panel on the left shows a normal sequence, and the one on the right depicts the sequence harbouring the point mutation at codon 277. (**B**) An 18-bp region in exon 5 was deleted in one cPNET. The sequence in the panel on the left depicts a normal sequence with nucleotides 13201–13218 highlighted, which are missing in the sample on the right.

**Figure 4:**
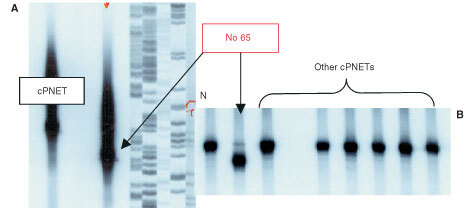
The autoradiographs of two acrylamide gels illustrate examples of the bands representing α^32^P-dATP-labelled PCR products generated from PCR of p53 exon 5. In (**A**), the band representing the truncated product from the cPNET (sample number 65) harbouring the deletion was viewed against that of another cPNET with a normal sized product and a sequencing ladder of a plasmid. In (**B**), the products from another cPNETs with a normal exon 5 were run alongside those from sample 65. A normal tissue control (N) was included in the reaction to generate a PCR product of the expected size for comparison.

DNA sequencing identified alterations in exon 5 of the p53 gene in three samples from immunostaining group A, including missense mutations in exon 5 of the p53 gene in two samples (Figure 3A

, B), and an in-frame deletion of 18 base pairs, corresponding to codons 275–280, in a third sample. This deletion was confirmed by PCR-direct size analysis (Figure 4A

, B) but no evidence of this deletion was found in the other 27 cases analysed by this method. A single base alteration in the DNA sequence of p53 was also detected in intron 6 in a sample which was negative for p53.

### Prognostic factors in cPNETs

**Figure 5:**
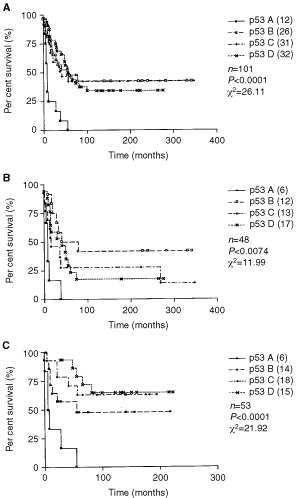
(**A**) Kaplan–Meier survival analysis found that expression of p53 had significant prognostic value in cPNETs, with a high intensity of DO-7 staining associated significantly with poorest survival (*P*<0.0001, log rank tested). When samples diagnosed before and after 1979 were analysed separately ((**B**) and (**C**) respectively), high intensity of DO-7 staining retained its prognostic significance (*P*=0.0074 and *P*<0.0001, respectively). *n*=number of samples; *P*=*P*-value; χ^2^=chi square value of the test.

The prognostic value of the accumulation of p53, Waf-1, Bax and Bcl-2 proteins was assessed by both univariate and multivariate analyses, using the Kaplan–Meier method and Cox's regression, respectively. The overall survival of the patients studied (*n*=101) was 35% at 5 years. The accumulation of p53 protein, which in the majority of cases was wild-type, has significant prognostic value (Figure 5

) in this series of cPNETs, with high SI (group A) relating to poor survival (*P*<0.0001, Log rank test). No significant difference in survival was observed between groups categorised for Waf-1, Bax and Bcl-2 staining. The categories and corresponding *P*-values for the survival analysis for each protein are shown in Table 3

**Table 3:**
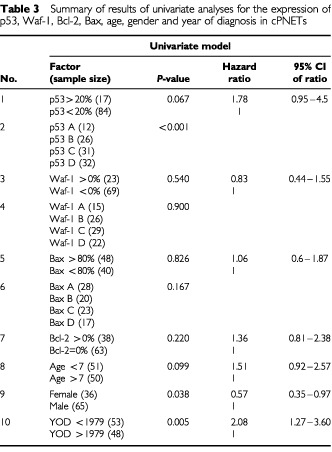
Summary of results of univariate analyses for the expression of p53, Waf-1, Bcl-2, Bax, age, gender and year of diagnosis in cPNETs

. Other factors, gender, age, and year of diagnosis, were also subjected to both univariate and multivariate analyses, and were found to be independently prognostic in the multivariate analyses (Table 4

**Table 4:**
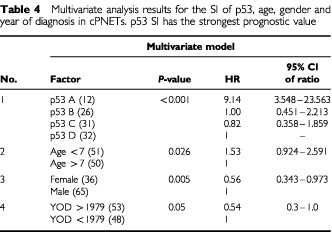
Multivariate analysis results for the SI of p53, age, gender and year of diagnosis in cPNETs. p53 SI has the strongest prognostic value

). However, of all the factors studied in the multivariate analyses, immunohistochemical staining of p53 had the strongest independent prognostic value.

## DISCUSSION

p53 protein expression was detected in the majority of the 101 cPNETs in this study, but only 12 of these (11.9%) had high staining intensity. The level of staining of p53 in the present study, which employed microwaving for antigen retrieval in the immunohistochemistry was consistent with that of a previous study (Jaros *et al*, 1993), in which no antigen retrieval was employed. DNA sequencing results indicated that p53 gene mutations in this series of cPNETs were infrequent, which was consistent with the findings from other studies (Saylors *et al*, 1991; Adesina, 1994) and could explain the high sensitivity of medulloblastoma cells to radio therapy (Chang *et al*, 1969).

The low incidence of p53 gene mutations in these cPNETs suggested that the p53 protein in these tumours was likely to be wild-type and functional. This was consistent with the significantly positive relationship between the expression of p53 and Bax (Table 5

**Table 5:**
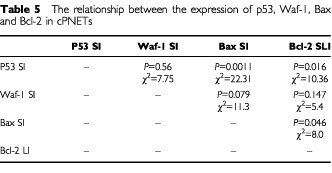
The relationship between the expression of p53, Waf-1, Bax and Bcl-2 in cPNETs

) which is a known target of p53 transcriptional activity (Miyashita and Reed, 1995). The expression of wild-type p53 in cPNETs may reflect a normal cellular response to the stressful microenvironment in the tumours, including nutrient deprivation, hypoxia and redox imbalance which would induce and stabilise WTp53 up-regulation (Graeber *et al*, 1994; An *et al*, 1998). Normal cells in such conditions would have undergone apoptosis, but it is likely that in some of these tumours, apoptosis was inhibited by the presence of Bcl-2. On the other hand, the expression of Waf-1 did not relate to that of p53 in these tumours (Table 5) which might not be unexpected, as Waf-1 expression is not exclusively controlled by p53 (Halevy *et al*, 1995; Russo *et al*, 1995; Liu *et al*, 1996). However, disturbance in the expression of Waf-1 protein in cells which over-express non-functional wild-type p53 has been reported in gliomas and glioma cell lines (Pykett *et al*, 1998) and the lack of relationship between the expression of these two proteins has also been reported in colorectal carcinoma (Slebos *et al*, 1996) and breast carcinoma (Barbareschi *et al*, 1996).

Contrary to p53 and Bax, the positive relationship between the expression of p53 and Bcl-2 proteins in the cPNETs (Table 5) was unexpected, as an inverse correlation between the expression of the two proteins would have been predicted, based on the current understanding of the suppressive function of p53 on Bcl-2 transcription (Seto *et al*, 1992; Miyashita *et al*, 1994a,b). This observation could be the result of induction of p53 in response to the elevated levels of Bcl-2, which could in turn be the result of a factor or mechanism that has given rise to the tumours. A likely candidate for this factor or mechanism was growth factor/receptor or oncogene de-regulation, as some of the tumours in this study have been found to over-express *erb*B2, a member of the epidermal growth factor receptor or EGFR family (Gilbertson *et al*, 1997, 1998).

In conclusion, the present study has shown that a high level of p53 protein in cPNETs measured by immunostaining intensity was associated with poor patient survival, supporting the findings of a previous study, and appeared to reflect the aggressiveness of the tumours. Sequencing results and the overall positive relationship between the expression of p53 and Bax pointed to the possibility that the p53 protein in the current series of cPNETs was wild-type and functional, except in the subset of tumours which expressed high levels of Bcl-2. In addition, maleness, young age at diagnosis and diagnosis before 1979 were all independent indicators of poor prognosis. Further research work, including functional assays involving reporter systems in cell lines and studies investigating the mechanisms underlying the expression of Bcl-2 in cPNETs, will clarify the functional status of p53 in central primitive neuroectodermal tumours.
